# Standardization of coagulometric tests in griffon vultures (*Gyps fulvus*): PT, aPTT and factor V reference values

**DOI:** 10.3389/fvets.2026.1761652

**Published:** 2026-05-05

**Authors:** Juan A. De Pablo-Moreno, Andrea Miguel-Batuecas, Jaime Carretero, Fernando González, Alberto Alvarado-Piqueras, Manuel Fuertes-Recuero, Jose Antonio García, Luis Revuelta

**Affiliations:** 1 Department of Veterinary Medicine, School of Biomedical and Health Sciences, Universidad Europea de Madrid, Villaviciosa de Odón, Spain; 2 Department of Physiology, School of Veterinary Medicine, Complutense University of Madrid, Madrid, Spain; 3 Department of Genetics, Physiology and Microbiology, School of Biological Sciences, Complutense University of Madrid, Madrid, Spain; 4 GREFA (Grupo de Rehabilitación de la Fauna Autóctona y su Hábitat), Majadahonda, Madrid, Spain; 5 Sección Departamental de Farmacología y Toxicología, Facultad de Veterinaria, Universidad Complutense de Madrid, Madrid, Spain; 6 Animal Health Department, School of Veterinary Medicine, Complutense University of Madrid, Madrid, Spain; 7 Department of Animal Medicine and Surgery, Veterinary Medicine School, Complutense University of Madrid, Madrid, Spain; 8 Complutense Veterinary Teaching Hospital, Complutense University of Madrid, Madrid, Spain

**Keywords:** avian coagulation diagnosis, birds of prey, coagulation factor V, coagulation time, griffon vultures

## Abstract

The avian coagulation system has structural and functional features hinder the direct application of commercial diagnostic products developed for humans. Griffon vultures (*Gyps fulvus*) are frequently admitted to wildlife rehabilitation centers with coagulation disorders due to lead or rodenticide poisoning. These disorders are challenging to diagnose due to the absence of reference intervals and validated coagulometric measurement protocols. The aim of this study was to standardize the measurement of prothrombin time (PT), activated partial thromboplastin time (aPTT), and coagulation factor V (FV) in griffon vultures using diagnostic reagents from human medicine, while also analyzing for differences according to sex, cause of admission, and weight. Plasma samples from 17 healthy individuals were analyzed using a semi-automated coagulometer and standard calibration reagents for human use. A specific protocol was established for the analysis of griffon vulture plasma using human diagnostic reagents adapted for the analysis. PT, INR, and FV could be successfully assessed under these conditions, allowing the establishment of reference intervals, whereas aPTT values were markedly prolonged and highly variable. These results allow for the establishment of a standardized protocol for accurately assessing coagulation in griffon vultures, which may serve as a reference for other avian species. The protocol could improve the clinical follow-up of cases of lead or rodenticide poisoning and ultimately contribute to wildlife conservation.

## 1 Introduction

Haemostasis is the physiological process by with the body responds to vascular injury (1–4). This process can be divided into two phases: primary haemostasis, which involves platelet activation, and secondary haemostasis, which comprises the extrinsic or tissue factor pathway, the intrinsic or amplification pathway, and the common pathway (3). These pathways are formed by specialized proteins called coagulation factors, which are found in blood plasma (3). The functionality of these pathways is assessed by measuring coagulation times. The extrinsic pathway is evaluated using the prothrombin time (PT), which also estimates the activity of factors VII, V, X, prothrombin, fibrinogen, and fibrin. The intrinsic pathway is evaluated using the activated partial thromboplastin time (aPTT), which also assesses the activity of factors XII, XI, X, IX, VIII, V, prothrombin, fibrinogen and fibrin (5, 6). Additionally, the international normalized ratio (INR) for PT and the ratio for aPTT can be used to standardize these measurements. All of these analytical tests and assessments of individual factor activity are performed on sodium citrate-treated plasma samples (6). However, the diagnostic performance and interpretation of PT and aPTT in avian species are highly dependent on the species and the method used. Recent studies on PT and aPTT at the point of care and viscoelastic studies emphasize the need for species-specific validation before extrapolating mammalian reference intervals or analyzing assay behavior in birds (7–9).

Factor V (FV), a commonly evaluated in coagulation studies, is a protein composed of several structural domains, including light and heavy chains linked by a large central B domain. FV plays a key role in haemostasis by participating in the common coagulation pathway, although it must first be activated (FVa) by cleavage of the B domain. Once activated, FV forms part of the prothrombinase complex alongside activated factor X (FXa), membrane phospholipids, and calcium ions (Ca^2^^+^), thereby catalyzing the conversion of prothrombin (FII) to thrombin (FIIa) (10).

Birds have a coagulation system similar to that of mammals (11), although avian coagulation times are typically longer (12–14). Genomic studies have revealed structural differences in avian coagulation factors, including prothrombin (FII) (13), and tissue factor (TF) (15–17), compared to mammals. Birds also lack the gene that encodes factor XII, and they retain a single combined copy of the prekallikrein-factor XI (PK-FXI) gene. In contrast, mammals have two separate genes for these factors (18–21). These structural differences, therefore, make it difficult to analyse coagulation using commercial reagents designed for humans, which are not optimized for use in birds (16). In birds, the extrinsic coagulation pathway, assessed through prothrombin time (PT), is considered the predominant and most clinically relevant mechanism, whereas the intrinsic pathway, evaluated by activated partial thromboplastin time (aPTT), appears to play a minor role with uncertain clinical significance (7). In birds, PT is generally considered to be the most clinically informative routine coagulation assay, as avian-specific differences in the contact pathway can complicate the interpretation of aPTT. However, recent studies have revealed significant variations in results across species and analytical platforms. This supports the idea of taking a cautious, validation-based approach rather than assuming that mammalian assay results can be applied universally (7–9).

The main causes of coagulopathies in birds are hepatic disorders (22), avian influenza virus infections (19, 23), disseminated intravascular coagulation (24) and rodenticide or possibly lead poisoning (25, 26). Reported PT alterations vary according to the underlying condition. In wild turkey poults exposed to aflatoxin, PT increased only slightly, from 10.24 ± 0.97 s in controls to 10.30 ± 0.80, 10.55 ± 0.50, and 10.76 ± 0.64 s depending on dose (22). In contrast, chickens with highly pathogenic H5N1 infection showed PT prolongations of 5.9 and 7.8 s above baseline at 12 and 24 h post-infection, respectively (23), while disseminated intravascular coagulation due to *Erysipelothrix rhusiopathiae* caused marked PT and APTT prolongation (PT 65.7 ± 14.4 s; APTT 194.6 ± 101.8 s vs. 50.7 ± 13.8 s and 82.4 ± 25.0 s in controls) (24). The primary cause of vitamin K deficiency in birds of prey is exposure to anticoagulant rodenticides used in agriculture. This inhibits the synthesis and activation of vitamin K-dependent coagulation factors (25, 27–30). Ingesting these rodenticides could result in the formation of haematomas, internal bleeding, hypovolaemic shock and death (27, 31, 32). In birds of prey, anticoagulant rodenticide exposure is the main cause of vitamin K deficiency and may severely prolong coagulation times, with mean PT values of 125.63 ± 110.44 s reported in poisoned hawks compared with 15.4 ± 2.9 s in healthy individuals (30). Recent studies in the field have increased concerns about the widespread secondary exposure of avian scavengers, including European scavenging species such as vultures, to second-generation anticoagulant rodenticides. These studies have also highlighted the potential for sublethal physiological effects. Meanwhile, current toxicological reviews emphasize that interpreting residues and establishing causality for coagulopathy or mortality is difficult because thresholds vary according to species, compound, exposure scenario and analyzed tissue (33–37).

Conversely, lead poisoning is more prevalent in scavenging birds of prey due to the ingestion of game remains containing lead bullet fragments (38, 39). Several studies in mammals have shown that lead affects erythrocytes, causing anemia and increasing the risk of thrombosis and cardiovascular problems (40–42). Therefore, a similar effect could be produced in avian species related to lead poisoning. Recent syntheses confirm that lead poisoning continues to represent a significant threat to raptors worldwide, with substantial implications for conservation and the One Health approach. Notably, recent evidence from Spain focusing on vultures has revealed that reducing hunting-related lead exposure can lower blood lead levels, even when residual exposure persists. This underscores the need for improved clinical monitoring tools in rehabilitation and conservation settings (43, 44).

These poisonings result in many scavenger birds of prey, including various vultures, being brought to wildlife recovery centers. However, the accurate diagnosis of coagulopathy in avian remains difficult, limiting therapeutic interventions and leading to elevated mortality rates and significant population declines (25, 26, 38, 45, 46). Currently, the lack of bird-specific reagents hinders the standardization of an optimal coagulation measurement method, as only reagents for human medicine are commercially available. Additionally, the absence of established reference values for avian coagulation parameters prevents a clear distinction between healthy individuals and those with underlying disorders. Therefore, accurate identification of bleeding disorders in these species is only possible through post-mortem examination. Therefore, despite recent advances in exposure surveillance and the development of new avian coagulation testing methods, standardized plasma-based coagulometric protocols and reference intervals for large scavenging birds of prey, such as griffon vultures, remain unavailable (7–9, 33, 47).

This study aimed to establish and standardize the coagulometric parameters of the griffon vulture (*Gyps fulvus*) by evaluating PT, aPTT, and FV, a common pathway component, and to define reference ranges. In addition, possible variations in these parameters were assessed according to sex, weight, and reason for admission to the rehabilitation center.

## 2 Material and methods

### 2.1 Study design

A study was carried out using plasma samples obtained from 17 adult griffon vultures (*Gyps fulvus*), all of which were considered clinically healthy at the time of blood collection (males, *n* = 10; females, *n* = 7). The griffon vultures came from various regions of Spain and were admitted to the Hospital of the Group for the Rehabilitation of Wild Fauna and its Habitat (*Grupo de Rehabilitación de la Fauna Autóctona y su Hábitat*; GREFA) either due to transfer by other centers of wildlife rehabilitation or because of clinical conditions (Supplementary Table 1). Blood was collected during routine veterinary examinations, which confirmed that the animals selected were clinically healthy and considered suitable for release. Demographic details for each vulture can be found in the Supplementary material.

### 2.2 Ethics approval

All procedures were carried out in accordance with the relevant guidelines and regulations. As all samples were obtained during routine veterinary diagnostic procedures, the approval from the Local Commission of Ethics in Animal Experimentation was not required in line with Spanish legal regulations (Royal Decree 53/2013 of 1 February and the European Directive EU/2010/63).

### 2.3 Sampling

Blood was collected into tubes containing 3.2% sodium citrate (1 part citrate to 9 parts blood) and immediately the tubes were then centrifuged at 2,500 x g for 15 min to obtain plasma. The plasma was aliquoted into labeled Eppendorf tubes, and 10 μL from each aliquot was used to create a pooled plasma sample. All samples were stored at −80 °C. Prior to coagulation tests, the plasma samples were thawed at room temperature for 30 min.

### 2.4 Coagulation tests

PT, aPTT and FV were analyzed in griffon vulture plasma using a viscosity-based mechanical coagulometer (Start Max II R, Diagnostica Stago S.A.S., Barcelona, Spain), in accordance with the manufacturer's instructions. The corresponding programmes for each parameter were strictly followed. Four cuvettes (Start 4 Cuve, Diagnostica Stago S.A.S., Barcelona, Spain) were loaded into the coagulometer, and metal spheres (Diagnostica Stago S.A.S., Barcelona, Spain) were subsequently added for each one. All reagents were reconstituted according to the manufacturer's instructions. Once all the reagents had been added to the sample in the cuvettes, the reaction was initiated automatically and completed upon clot formation. The result, expressed in seconds, indicated the time required for clotting. Positive and negative controls for the reagents were included using the System Control N/P reagent (Diagnostica Stago S.A.S., Barcelona, Spain). This methodology was based on that described by De Pablo-Moreno et al. (14).

### 2.5 Prothrombin time analysis

PT was assessed using Neoplastin Cl+5 (Diagnostica Stago, Barcelona, Spain), which has an ISI (International Sensitivity Index) of 1.15. The reagent was reconstituted and pre-warmed at 37 °C prior to use. Metal spheres were placed in each well and pre-warmed at 37 °C for 3 min. Then, 50 μL of plasma sample was added to each well. After 60 s of incubation, 100 μL of Neoplastin Cl+5 was added to each well. The Owren Koller buffer was allowed to stand for 30 min before being used for dilution. PT values from individual samples were expressed in seconds, as percentages, and as international normalized ratio (INR) values. The INR was calculated as the ratio between the sample PT and a reference PT plasma value (generated by mixing all individual aliquots previously stored at −80 °C), raised to the power of the ISI.

First, a pool of citrated plasma from griffon vultures was analyzed using 1/1 (no dilution), 1/2, 1/3, 1/4, 1/6, 1/9 and 1/12 dilutions. These were compared with commercial calibrator measurements at 1/1, 1/2, 1/3 and 1/4 dilutions. For the calibration curves, a calibration curve was first generated using the commercial calibrator (Unicalibrator) and then compared with those generated from previously obtained results from the serial dilutions of the griffon vulture plasma pool. These latter curves were generated by two approaches: replicating the standard procedure for the commercial calibrator (1/1, 1/2, 1/3 and 1/4 dilutions) and adapting the dilutions to obtain similar slopes and ranges between dilutions (1/3, 1/6, 1/9 and 1/12 dilutions). Finally, the 1/1 and 1/3 dilutions were used to analyse the individual times of the vulture samples.

### 2.6 Factor V analysis

To assess FV levels, FV-deficient plasma (Sta-Deficient V, Diagnostica Stago, Barcelona, Spain) and Neoplastin Cl+5 (Diagnostica Stago, Barcelona, Spain) with an ISI of 1.15 were used. Metal spheres were placed in the cuvettes and pre-warmed at 37 °C for 3 min. Once reconstituted, the Neoplastin Cl+5 solution was homogenized and pre-warmed at 37 °C for 3 min before use. Owren Koller buffer (Diagnostica Stago S.A.S., Barcelona, Spain) was allowed to reach room temperature (30 min) before dilution. Then, 50 μL of FV-deficient plasma and 50 μL of the sample to be analyzed, previously diluted 1/10 in Owren Koller buffer, were added to the cuvette. After a 60-s incubation period, 100 μL of Neoplastin Cl+5 was added to initiate the reaction. Results were expressed in seconds and as percentages.

The griffon vulture plasma pools were analyzed first using 1/10, 1/20, 1/40 and 1/80 dilutions, and the results were compared with those of the commercial calibrator at the same dilutions.

For calibration, a calibration curve was generated using a commercial calibrator (Unicalibrator) and this was compared with those generated from previously obtained results from the serial dilutions of the griffon vulture plasma pool using the same procedure recommended for the commercial calibrator (1/10, 1/20, 1/40 and 1/80 dilutions). For the determination of individual FV times, a 1/10 dilution was used.

### 2.7 Activated partial thromboplastin time analysis

The aPTT was assessed by placing metal spheres into all cuvettes and pre-warming them at 37 °C for 3 min. Then, 50 μL of an undiluted plasma sample and 50 μL of PPT Automate reagent (Diagnostica Stago S.A.S., Barcelona, Spain) were added to each cuvette. After incubating the mixture for 180 s, 50 μL of 0.025 M CaCl_2_ (Diagnostica Stago S.A.S., Barcelona, Spain) was added. Results were expressed in seconds and as the ratio of the sample value to that of the plasma pool. The griffon vulture plasma pool was analyzed at a 1/1 dilution, and the results were compared with measurements from a positive control at the same dilution. No calibration curve was required due to international standards.

### 2.8 Reference intervals

Statistical analysis was performed using the SPSS version 27 statistical package (version 9.4; SAS Institute, Cary, NC, USA), and calibration curves were created using Excel (Microsoft Office 365). The Shapiro–Wilk test was applied to assess the normality of data distribution. An analysis of covariance (ANCOVA) was performed to evaluate the influence of weight (included as a covariate) and assess the effects of sex and cause of admission on clotting times and FV levels. Common descriptive statistics for the variables FV, PT, and aPTT was performed including minimum and maximum value, mean ± 2 standard deviations (SD), coefficient of variation (%) and median. Results were considered statistically significant at *P* < 0.050.

## 3 Results

### 3.1 Calibration study

To determine the optimal dilution for the PT and FV calibration curves, multiple measurements were performed at different dilutions using a pool of citrated plasma samples from 17 individuals, alongside a commercial calibrator. Similarly, aPTT was compared with a positive control (Table 1).

**Table 1:** Prothrombin time (PT), factor V (FV) and activated partial thromboplastin time (aPTT) measurements at different proposed dilutions.

PT			FV			aPTT		
Dilution	Calibrator (seconds)	Pool (seconds)	Dilution	Calibrator (seconds)	Pool (seconds)	Dilution	Positive control (seconds)	Pool (seconds)
1/1	13.9	48.0	1/10	27.0	26.7	1/1	36.4	186.5
1/2	20.7	50.0	1/20	31.5	30.1			
1/3	30.3	55.2	1/40	39.5	35.7			
1/4	39.2	60.7	1/80	46.6	40.5			
1/6		72.5						
1/9		93.7						
1/12		117.7						

Significant differences were observed in the PT measurements between the griffon vulture plasma pool and the commercial calibrator. The vulture samples showed longer clotting times at equivalent dilutions. Furthermore, the initial dilutions showed shorter time ranges, which progressively widened with increasing dilution. In contrast, FV measurements from the vulture plasma pool were very similar to those of the commercial calibrator, particularly at the lower dilutions (1/10 and 1/20). Regarding aPTT, the differences were even more pronounced, with substantially longer clotting times observed in the animal plasma pool compared to the positive control.

Calibration curves were generated for the PT and FV parameters using the commercially available calibrator and griffon vulture plasma pool, starting from the most appropriate initial dilution. PT and FV percentages were calculated from the clotting times obtained in seconds for each test. The distances between dilution points and the slopes of the resulting curves were analyzed.

**Figure 1:**
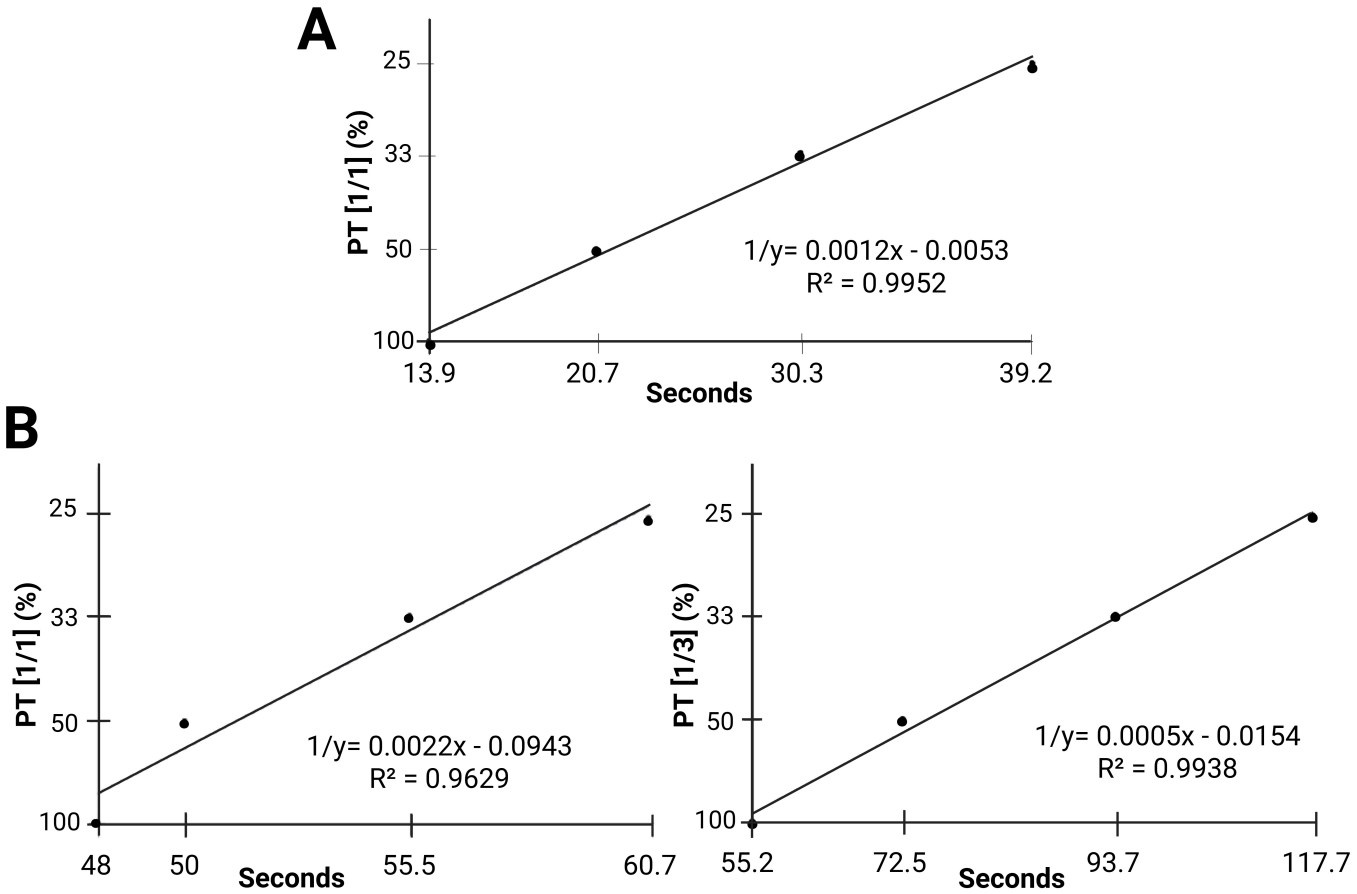
PT calibration curves: **(A)** PT curve generated using the commercially available commercial calibrator. **(B)** PT curve generated using the vulture plasma pool (sample obtained by mixing plasma from all individuals) at a 1/1 **(left)** and a 1/3 **(right)** dilutions as the first measurement point of the calibration curve. Abbreviations: PT: prothrombin time.

In the PT calibration curves (Figure 1), differences were observed between the commercial human calibrator and the vulture plasma pool. The 1/1 dilution curve from the vulture pool (Figure 1B) had a higher slope (0.0022); however, the time intervals between dilutions were short (approximately 2 s), and an R^2^ of 0.96 indicated lower robustness. This suggested that even minor technical variations could lead to substantial deviations. In contrast, the curve generated with the 1/3 dilution had a lower slope (0.0005) but displayed longer time intervals between dilutions. This suggested greater stability, robustness, and similarity to the curve generated with the commercial calibrator. The higher R^2^ value further supports the greater precision, minimizing the impact of possible technical variations in the measurement.

**Figure 2:**
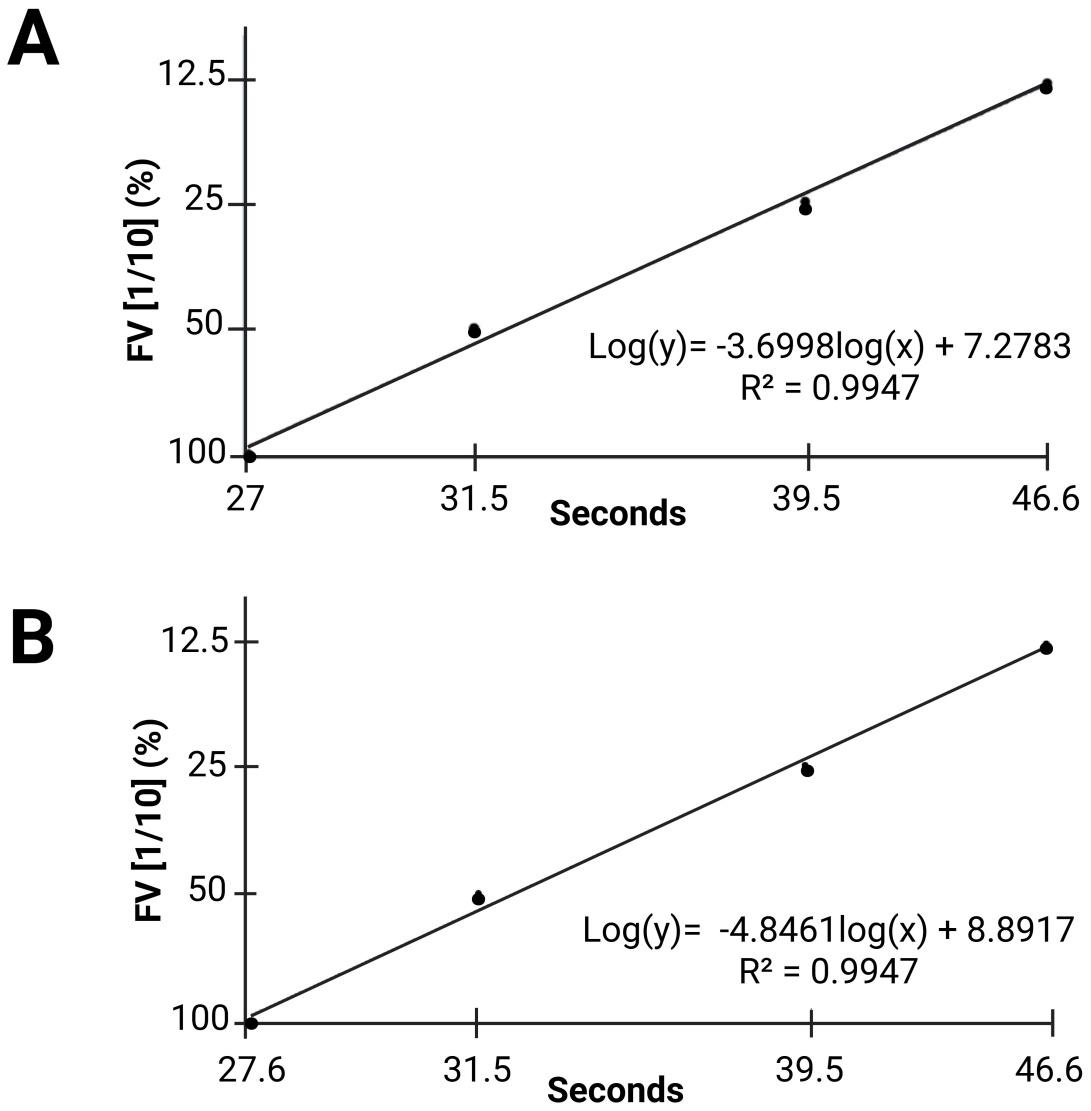
FV calibration curves: **(A)** FV curve generated using the commercially available calibrator. **(B)** FV curve generated using the vulture plasma pool (sample obtained by mixing plasma from all individuals) at a dilution of 1/10 as the first measurement point of the calibration curve. Abbreviations: FV: factor V.

In the FV calibration curves (Figure 2), both the commercial calibrator and the vulture plasma pool displayed very similar behavior in terms of the slope and the measurement range across the various dilutions. Additionally, both curves had an R^2^ value of almost 1, indicating high comparability between the initial points of the dilution range and stable responses without variation. The profiles obtained in both cases are almost identical, which reinforces the reliability and reproducibility of the measurements.

Finally, no calibration curve was required for aPTT, as results are expressed solely in seconds in accordance with international standards. Compared with the commercial control, aPTT measurements were up to five times longer.

### 3.2 Statistical study

A descriptive analysis was conducted to assess whether the possible influence of sex and cause of admission (Supplementary Table 1) to the center on the weight of the animals, using ANCOVA, with the PT, FV and aPTT variables. By sex, *n* = 10 individuals were male (58.8%) and *n* = 7 were female (41.2%). Mean body weight was 8,194 ± 704.9 g. Regarding cause of admission, *n* = 6 vultures had been admitted due to lead poisoning after recovery (35.3%), *n* = 2 due to trauma after recovery (11.8%), and *n* = 9 were transferred from another rehabilitation center (52.9%).

The Shapiro-Wilk test (*P* > 0.050) indicated a normal distribution for all coagulometric variables. Statistically significant differences were observed in the aPTT values with respect to the cause of admission (*P* = 0.033), with no influence of weight. The mean aPTT values for vultures admitted due to pathology was 268.06 s, compared to 183.27 s for those transferred from another center.

**Figure 3:**
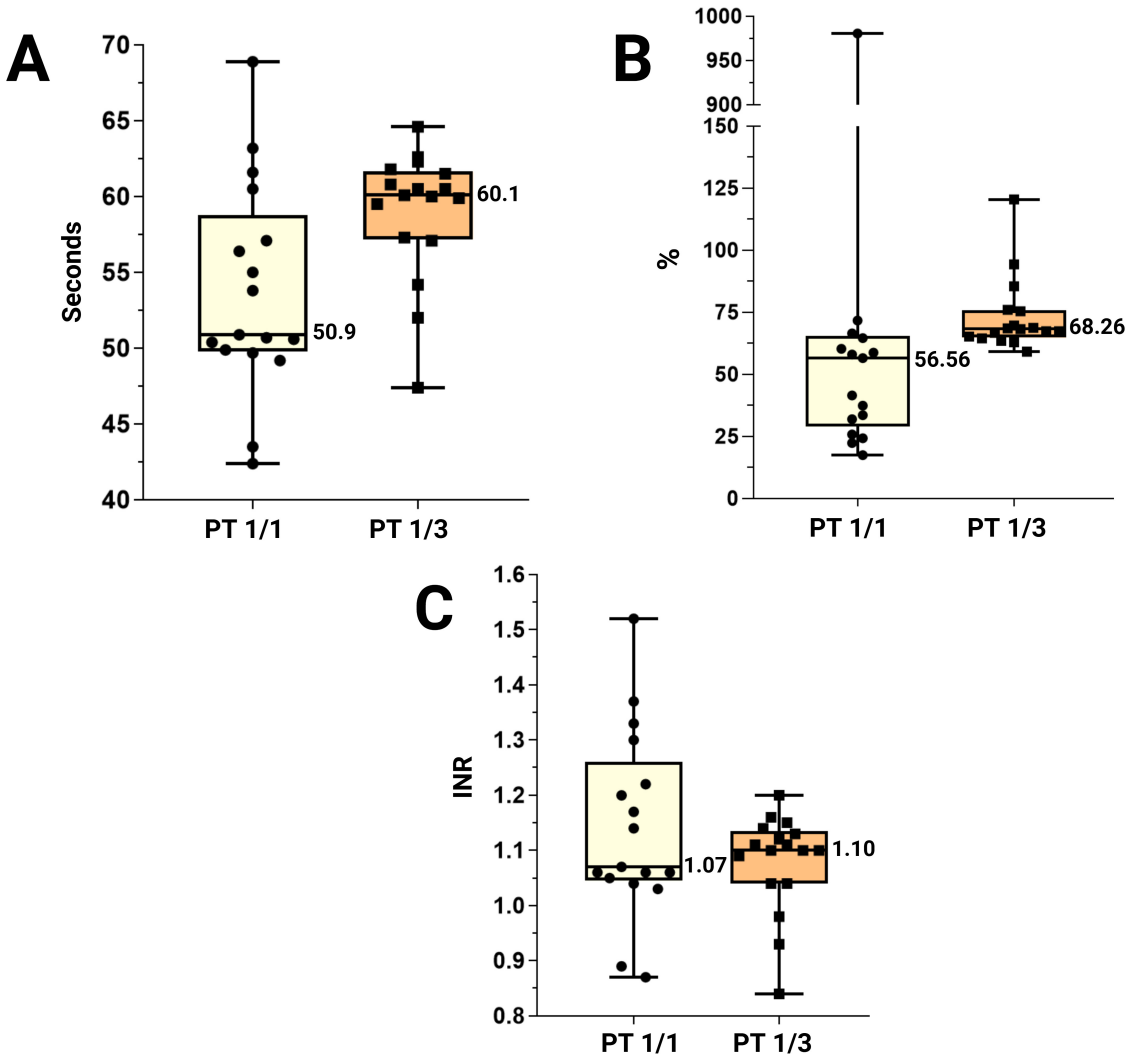
Representation of PT values, indicating the maximum and minimum values. **(A)** PT 1/1 and PT 1/3 expressed in seconds. **(B)** PT 1/1 and PT 1/3 expressed as percentage. **(C)** The INR values corresponding to PT 1/1 and PT 1/3. Abbreviations: INR: International Normalized Ratio; PT: prothrombin time.

For PT (Figure 3), the mean ± 2SD at a 1/1 dilution was 139.14% ± 543, with a median of 56.56 (53.75 ± 13.88 s; median 50.9). For the 1/3 dilution, the mean ± 2SD was 73.15% ± 29.90, with a median of 68.26 (58.94 s ± 8.56; median 60.1). PT data for both dilutions were also expressed as INR, using the pooled plasma sample as a reference: 48.0 s for the 1/1 dilution and 55.2 s at a 1/3 dilution. The resulting mean ± 2SD INR values were 1.14 ± 0.34 and 1.08 ± 0.18, respectively, with corresponding medians of 1.10 and 1.07.

**Figure 4:**
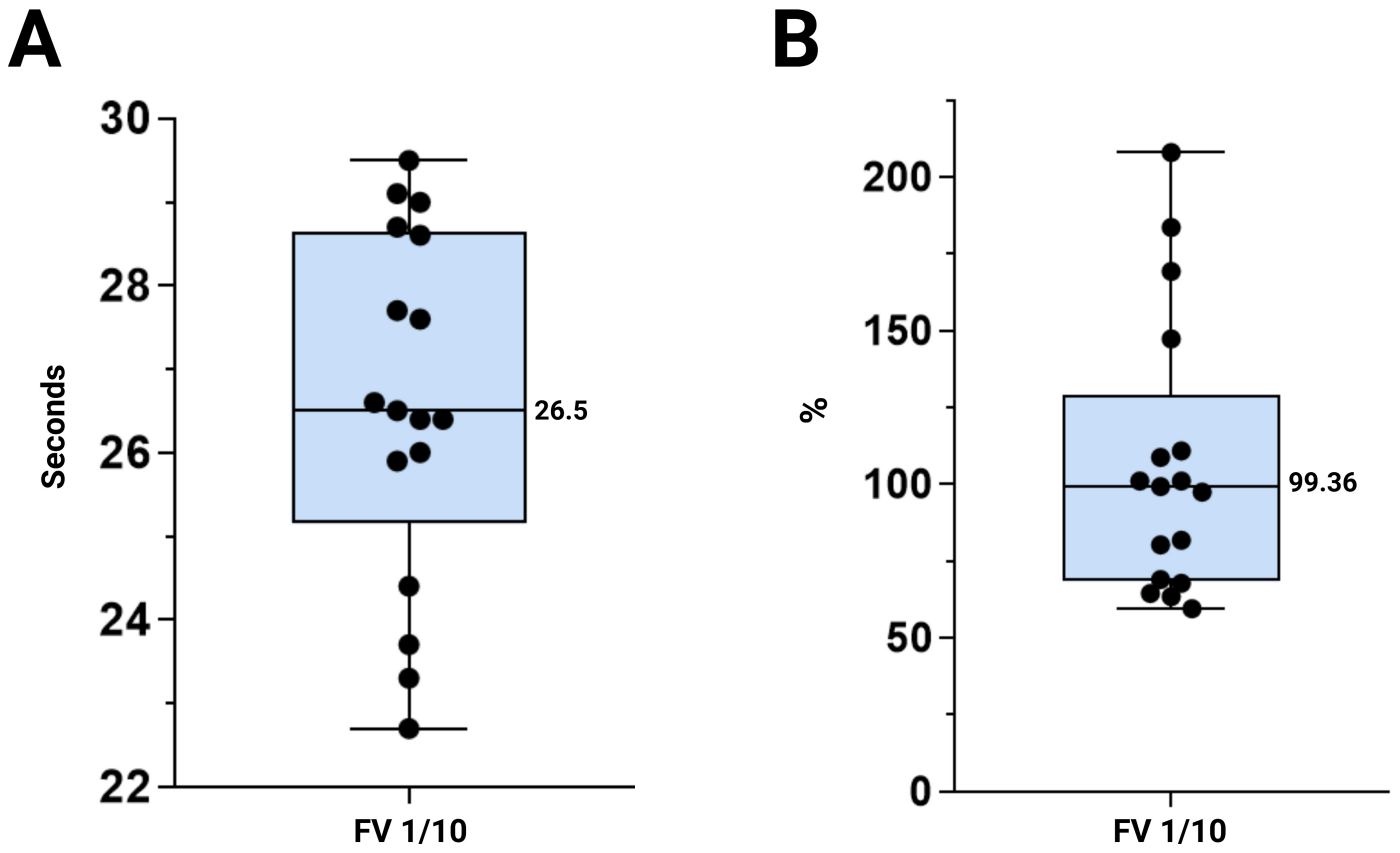
Representation of FV values, indicating the maximum and minimum values. **(A)** FV 1/10 expressed in seconds. **(B)** FV 1/10 expressed as a percentage. Abbreviations: FV: factor V.

**Figure 5:**
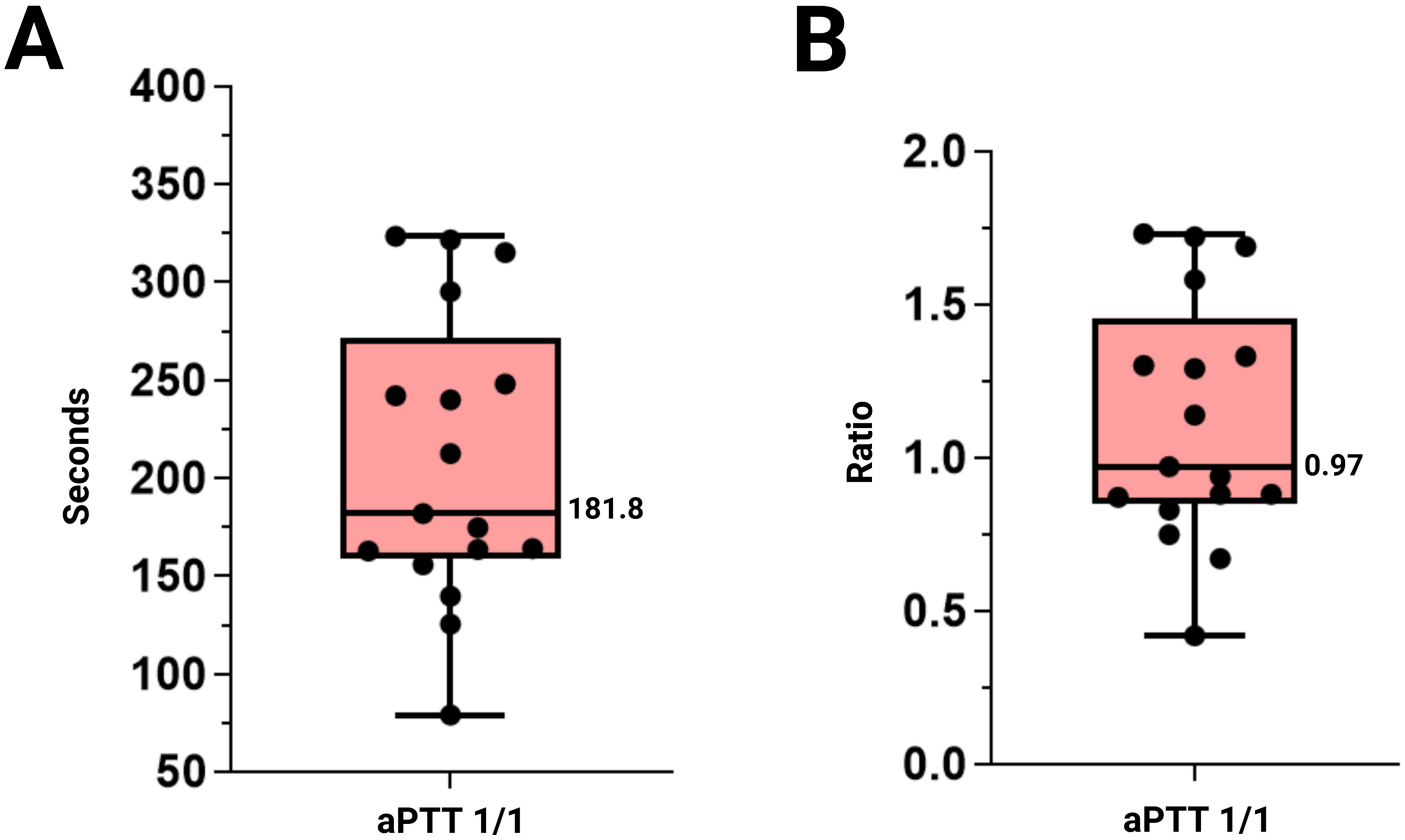
Representation of aPTT values, indicating the maximum and minimum values. **(A)** aPTT 1/1, expressed in seconds. **(B)** aPTT expressed as a ratio. Abbreviations: aPTT: Activated partial thromboplastin time.

The mean ± 2SD FV at a 1/10 dilution was 106.72% ± 89.60, with a median of 99.36 (26.59 s ± 4.22; median 26.5) (Figure 4). aPTT at a 1/1 dilution were 208.42 ± 148.22 s, with a median of 181.8 s. The corresponding ratio was 1.12 ± 0.79, with a median of 0.97 (Figure 5). Descriptive statistics expressed as minimum, maximum, mean ± SD, coefficient of variation and median are shown in Table 2. Individual measurements are provided in the Supplementary material.

**Table 2:** Common descriptive statistics for the variables FV, PT, and aPTT.

Variable	Minimum value	Maximum value	Mean ±2 standard deviations (SD)	Coefficient of variation (%)	Median
%FV [1/10]^*^	59.54	208.09	106.72 ± 89.60	83.96	99.36
FV [1/10] (sec)	22.70	29.50	26.59 ± 4.22	15.87	26.50
% PT [1/1]	17.46	980.39	139.14 ± 543	390.25	56.56
PT [1/1] (sec)	42.40	68.90	53.75 ± 13.88	25.82	50.90
% PT [1/3]	59.17	120.63	73.15 ± 29.90	40.86	62.86
PT [1/3] (sec)	47.40	64.60	58.94 ± 8.56	14.52	60.10
INR [1/1]	0.87	1.52	1.14 ± 0.34	29.81	1.07
INR [1/3]	0.84	2.20	1.07 ± 0.18	35.18	1.01
aPTT [1/1]	78.80	323.50	208.42 ± 148.22	71.11	181.80
Ratio	0.42	1.73	1.12 ± 0.79	35.55	0.97

^*^The numbers in brackets indicate the dilution of the sample.

## 4 Discussion

The coagulation system is a complex cascade of enzymatic reactions involving a large number of components that can be disrupted for different causes, such as lead or rodenticide poisoning (2, 3), a common occurrence in scavenging birds of prey (25, 26, 32, 40, 45, 48, 49). However, assessing the coagulation system in birds using coagulometric testing is challenging due to the absence of validated protocols and significant structural and functional differences compared to mammalian haemostatic systems. These differences may affect the reactivity of reagents designed for humans without necessarily indicating dysfunction to the species being tested. This limitation may lead to the misinterpretation of results, compromising diagnosis and the implementation of appropriate treatment in affected species (50). In this study, we analyzed and standardized PT and aPTT coagulometric values and FV levels in griffon vultures (*Gyps fulvus*) using a coagulometer that is routinely employed in clinical practice.

PT values obtained at various dilutions were prolonged compared to mammals. Previous studies have reported similar results in chickens (dilution 1/2) and American flamingos (dilution unspecified), although data dispersion was high (16, 17). In contrast to our calcium-thromboplastin PT assay, some studies have used Quick's method with crude chicken hatchling thromboplastin (CHT), yielding shorter PTs that are reported as clotting time in seconds, not as % activity, and without a standard calibration curve (51). The calibration curve generated using 1/1, 1/2, 1/3 and 1/4 dilutions, showed only 2-s difference (from 48 to 50 s) between the first and second dilutions. This suggests that minor timing variations can result in substantial differences in PT percentage values. Therefore, a 2-s difference could result in a 50% discrepancy, potentially misclassifying healthy individuals with slight variations in coagulation times as unhealthy (14).

This limitation was overcome by adapting a calibration curve in accordance with the methodology of De Pablo-Moreno et al. (14), using 1/3 dilution as the reference point for 100%, 1/6 as 50%, 1/9 as 33% and 1/12 as 25%. This resulted in a difference of 17.3 s between the 1/3 and 1/6 dilutions, ensuring that minor differences in seconds do not lead to significant changes in measured values in PT percentage. The mean values for the individual PT times were 53.75 and 58.94 s for the 1/1 and 1/3 dilutions, respectively. These values were considerably longer than human reference times (10–14 s) but 18.93% below the reference values for other wild birds, such as the American flamingo (72.7 s) (17).

The use of INR in avian coagulation analysis had not previously been proposed, but it could potentially serve as a future reference parameter for PT measurements in birds. In humans, INR reference values are between 0.8 and 1.2 (14, 52, 53). In this study, the most accurate values were obtained from PT measurements at a dilution of 1/3, showing a lower dispersion of the parameters and a mean closer to 1.

Conversely, the mean values obtained for FV were 106.72%, which lies within the reference range for humans (50–200%) (54). These results may be due to the use of a commercial FV deficient reagent, despite the excess presence of all other factors. As this reagent includes the human extrinsic pathway activators and is designed for human plasma, the pronounced PT prolongation observed in birds was not seen in the FV analysis. This suggests that the PT prolongation clotting time in birds is due to factors acting before FV. Previous studies have proposed that the issue does not stem from lower concentrations of factors, such as V, VII or X, but rather that from structural differences. Mammalian tissue factor (TF) reagent is unlikely to react well with FVII in bird plasma when measuring the PT, making it difficult to form fully active enzyme complexes and initiate the extrinsic pathway (15–17).

Given that the values of FV obtained in the study were within the range expected for humans and differed from the prolongation observed in PT. This indicates that the functional activity of FV in birds, when assessed using commercial reagents, is comparable to that in humans. Although no specific characterization of FV in Griffon vultures is available, studies in golden eagle (*Aquila chrysaetos*) have shown that there is a strong conservation of sequences in the A1, A2, A3, C1 and C2 domains, with the B domain displaying the highest variability (55). This suggests that FV in griffon vultures may interact effectively with human reagents, thus explaining the consistency of the obtained values.

Finally, the most widely dispersed value was the aPTT clotting time, with a mean value of 208.43 s, which is higher than the reference range for human (25–45 s) and 213.76% higher than the reference range for other birds, such as flamingos (97.5 s) (17). These prolonged aPTT times are consistent with the previously described clotting times in the intrinsic coagulation pathway in birds compared to mammals. Birds lack the gene encoding Factor XII, the pathway is primarily initiated by FIX, which is activated by the FXa-FVIIa complex. To a lesser extent, it is also started by FXI activation following contact with a negatively charged surfaces, such as kaolin and silica, which are used in routine coagulometry (18, 21). The ratio is used to reduce dispersion and improve comparability between laboratories and species, similar to the INR for PT. This strategy is already employed for anticoagulated patients to minimize variability arising from differences in protocols, operators, techniques, and reagents (56).

Furthermore, analysis data analysis showed no relationship between sex. However, previous studies in other species, including humans, have observed differences in platelet aggregation and clotting factor levels between sexes, primarily due to the influence of sex hormones (estrogen and testosterone) (20, 57–59). Therefore, these differences were not clinically significant. The absence of differences could be attributed to the small sample size (*n*) compared to human clinical studies, which analyse larger numbers of patients. It is plausible that the avian coagulation cascade, which shows substantial evolutionary conservation, may exhibit limited sex-related hormonal modulation; however, direct evidence in birds is currently lacking and coagulation assays (particularly aPTT) remain only partially validated (7, 21). Regarding the cause of admission in the center and aPTT, a significant relationship was identified. Notably, cases involving sequelae should have shown lower coagulation times for vultures admitted with lead poisoning due to lead has procoagulant effect, as it has been described in human medicine (40). However, this was not considered clinically relevant. Therefore, the results were analyzed together descriptively.

Regarding weight, while some studies have reported a correlation between weight and coagulation, this association has only been observed in cases of obesity. In these cases, chronic inflammation in the body activates prothrombotic signaling pathways through inflammatory cytokines, thereby increasing thrombin synthesis and platelet aggregation (60). In our study, all analyzed individuals were within the normal weight range for their age (6–10 kg), which may explain the absence of clinically significant differences (61).

One of the main limitations of the study is the small sample size. In contrast to domestic or laboratory animals, the difficulty of working with large samples is a characteristic of wildlife studies as inclusion depends on admissions for medical reasons that are beyond the control of the researcher.

Nevertheless, the results of this study support the presence of structural differences between avian and mammalian coagulation systems. However, much remains to be learned about the haemostatic system in birds. Further research could build on this by identifying an activator compatible with commercial reagents and by conducting future studies that assess changes in coagulation. This could facilitate the development of treatments for avian diseases, particularly those most affected by human activity, and enable meaningful contributions to their conservation and management through coagulation monitoring.

This study established an optimal protocol for measuring coagulation parameters in griffon vultures (*Gyps fulvus*) using commercially available reagents. The 1/3 dilution was found to be most suitable for evaluating prothrombin time (PT), while the 1/10 dilution was considered optimal for measuring factor V (FV). As no statistically significant differences in coagulation values were observed according to sex or weight, reference values were proposed for the species, expressed in seconds, percentage and international normalized ratio (INR) for PT, and in seconds and percentage for FV. For activated partial thromboplastin time (aPTT), reference values were expressed in seconds and as a ratio. These results allow a standardized protocol to be established for coagulometric assessment in griffon vultures using commercial reagents designed for human use. This protocol may serve as a reference for future research and its adaptation to other avian species. Moreover, applying this protocol in monitoring and diagnosis coagulation disorders, including intoxication caused by heavy metals or rodenticides, may represent a valuable tool for the conservation and management of wild birds.

## Data Availability

The original contributions presented in the study are included in the article/Supplementary material, further inquiries can be directed to the corresponding author.
