# Supplementary material for: Standardization of coagulometric tests in griffon vultures (Gyps fulvus): PT, aPTT and factor V reference values
Source: Front Vet Sci. 2026 May 5;13:1761652. doi: 10.3389/fvets.2026.1761652 (PMC13183543; doi:10.3389/fvets.2026.1761652)
Supplement: Supplementary file 1 [file Supplementary_file_1.docx]

Supplementary Material

**Supplementary Table 1.** Data on griffon vultures submitted for the study.

| **Identification** | **Sex** | **Location where they were found** | **Cause of admission** | **Weight (g)** |
| --- | --- | --- | --- | --- |
| 21/6611 | Male | San Martín de Valdeiglesias | Poisoning - Lead | 7700 |
| 21/7302 | Female | Zaidín | Transferred from another centre | 6900 |
| 21/7296 | Male | Peñalba | Transferred from another centre | 8300 |
| 21/7289 | Female | Calomarde | Transferred from another centre | 9300 |
| 21/7308 | Female | Pastriz | Transferred from another centre | 9200 |
| 21/6569 | Male | Manzanares el Real | Poisoning - Lead | 8400 |
| 21/7290 | Male | Bierge | Transferred from another centre | 8300 |
| 21/7292 | Female | Avizanda | Transferred from another centre | 8400 |
| 21/7300 | Male | Sallent de Gállego | Transferred from another centre | 8600 |
| 21/7291 | Female | Torrelisa | Transferred from another centre | 9200 |
| 21/7298 | Male | Jaca | Transferred from another centre | 8200 |
| 21/6988 | Male | El Boalo | Poisoning - Lead | 8000 |
| 21/6011 | Male | Guadarrama | Poisoning - Lead | 7800 |
| 21/3023 | Male | Galapagar | Trauma | 8500 |
| 21/6668 | Female | San Martín de Valdeiglesias | Poisoning - Lead | 8000 |
| 21/6610 | Female | Robledo de Chavela | Trauma | 6800 |
| 21/6869 | Male | Valdemanco | Poisoning - Lead | 7700 |

**Supplementary Table 2.** *Individual measurements of PT, FV and aPTT.*

| **Identification** | **PT 1/1 (s)** | **PT %** | **INR** | **PT 1/3 (s)** | **PT %** | **INR** | **FV (s)** | **FV %** | **aPTT** | **Ratio** |
| --- | --- | --- | --- | --- | --- | --- | --- | --- | --- | --- |
| 21/6611 | 50.9 | 56.56 | 1.07 | 57.3 | 75.47 | 1.04 | 26 | 108.83 | 163.9 | 0.88 |
| 21/7302 | 63.2 | 22.35 | 1.37 | 59.9 | 68.73 | 1.10 | 28.6 | 69.03 | 242.1 | 1.30 |
| 21/7296 | 49.9 | 64.60 | 1.05 | 54.2 | 85.47 | 0.98 | 23.3 | 183.72 | 155.6 | 0.83 |
| 21/7289 | 61.6 | 24.26 | 1.33 | 57.1 | 76.05 | 1.04 | 26.4 | 101.17 | 181.8 | 0.97 |
| 21/7303 | 56.4 | 33.58 | 1.20 | 59.5 | 69.69 | 1.09 | 24.4 | 147.39 | 125.2 | 0.67 |
| 21/6569 | 68.9 | 17.46 | 1.52 | 64.6 | 59.17 | 1.20 | 29 | 64.60 | 163.5 | 0.88 |
| 21/7290 | 43.5 | 714.29 | 0.89 | 47.4 | 120.48 | 0.84 | 23.7 | 169.37 | 139.5 | 0.75 |
| 21/7292 | 57.1 | 31.93 | 1.22 | 60.5 | 67.34 | 1.11 | 27.6 | 81.82 | 247.9 | 1.33 |
| 21/7300 | 49.2 | 71.74 | 1.03 | 62.3 | 63.49 | 1.15 | 29.5 | 59.54 | 162.6 | 0.87 |
| 21/7291 | 42.4 | 980.39 | 0.87 | 52 | 94.34 | 0.93 | 22.7 | 208.09 | 78.8 | 0.42 |
| 21/7299 | 50.4 | 60.31 | 1.06 | 60.5 | 67.34 | 1.11 | 28.7 | 67.89 | 212.5 | 1.14 |
| 21/6988 | 50.6 | 58.75 | 1.06 | 60.8 | 66.67 | 1.12 | 26.4 | 101.17 | 240 | 1.29 |
| 21/6011 | 53.8 | 41.56 | 1.14 | 61.5 | 65.15 | 1.13 | 27.7 | 80.42 | 321.6 | 1.72 |
| 21/3023 | 55 | 37.45 | 1.17 | 62.6 | 62.89 | 1.16 | 26.6 | 97.59 | 315 | 1.69 |
| 21/6668 | 60.5 | 25.77 | 1.30 | 61.8 | 64.52 | 1.14 | 29.1 | 63.55 | 323.5 | 1.73 |
| 21/6610 | 49.7 | 66.49 | 1.04 | 60.1 | 68.26 | 1.10 | 25.9 | 110.85 | 174.6 | 0.94 |
| 21/6869 | 50.7 | 58.00 | 1.06 | 60 | 68.49 | 1.10 | 26.5 | 99.36 | 295.2 | 1.58 |
| **Pool** | 48 |  |  | 55.2 |  |  | 26.7 |  | 186.5 |  |
